# Delirium superimposed on dementia in nursing homes: prevalence, incidence, and prognosis – protocol of a prospective multicenter cohort study

**DOI:** 10.1186/s12877-026-08038-w

**Published:** 2026-08-15

**Authors:** Vincent Molitor, Johanna Christina Seiters, Burkhard Haastert, Nina Willerscheidt, Imke Aits, Tim Stuckenschneider, Falk Hoffmann, Michael H Freitag, Tania Zieschang, Rebecca Palm

**Affiliations:** 1 Nursing Science, Department for Health Services Research, School of Medicine and Health Sciences, Carl von Ossietzky Universität Oldenburg, Ammerländer Heerstraße 114-118, Oldenburg, 26129 Germany; 2 mediStatistica, Wuppertal, Germany; 3 General Practice/Family Medicine, Department for Health Services Research, School of Medicine and Health Sciences, Carl von Ossietzky Universität Oldenburg, Oldenburg, Germany; 4 Geriatric Medicine, Department for Health Services Research, School of Medicine and Health Sciences, Carl von Ossietzky Universität Oldenburg, Oldenburg, Germany; 5 Pharmacoepidemiology and Outpatient Care, Department for Health Services Research, School of Medicine and Health Sciences, Carl von Ossietzky Universität Oldenburg, Oldenburg, Germany

**Keywords:** Longterm care, Epidemiology, Clinical outcomes, Confusion, Cognitive decline

## Abstract

**Background:**

Delirium is a frequent and serious neuropsychiatric syndrome in older adults. People living with dementia are at particularly high risk of developing delirium. In nursing homes, where dementia prevalence is high, delirium superimposed on dementia (DSD) represents a major clinical challenge and research gap. Aim of the study is to produce reliable data on prevalence and incidence of DSD in nursing homes as well as to investigate risk factors, clinical course, physical activity patterns, and care processes.

**Methods:**

This prospective multicenter cohort study will be conducted between August 2025 and July 2028 in three regions in northwestern Germany. Approximately 300 residents with dementia from about 20 nursing homes will be enrolled. Participants without DSD at baseline will undergo weekly delirium screening over six months using the Informant Assessment of Geriatric Delirium (I-AGeD), administered by trained research staff. In cases of positive screening, delirium severity and motor subtype will be assessed using the Delirium Rating Scale-Revised-98 (DRS-R-98) and Delirium Motor Subtype Scale (DMSS). Nursing and medical care processes related to delirium will be documented upon first occurrence of potential DSD. Physical activity will be assessed at baseline and reassessed at the first occurrence of potential DSD or, in participants without delirium, at the end of the six-month observation period. Prevalence and incidence of delirium will be estimated with adjustment for clustering at the nursing home level. Risk factors will be analyzed using multiple mixed logistic regression models, and time-to-event analyses will be applied for incident delirium. Explorative prognostic analyses will examine associations with cognitive decline, functional status, mortality, and physical activity.

**Discussion:**

Findings will inform development of context-sensitive prevention and management strategies to strengthen evidence-based delirium care in nursing homes.

**Trial registration:**

Prospectively registered on 17.04.2026 in the Deutsches Register für Klinische Studien (DRKS), (DRKS00039239; Date of registration in DRKS: 2026-04-17).

## Background

Delirium is an acute neuropsychiatric syndrome characterized by disturbances in attention, awareness, and cognition, frequently accompanied by perceptual disturbances, psychotic symptoms, and affective or behavioral changes such as agitation or hallucinations. A hallmark feature of delirium is its acute onset and fluctuating course, with symptom severity varying over short periods of time [1]. Pathophysiologically, delirium represents a phenotypic manifestation of acute encephalopathy, reflecting global and typically reversible cerebral dysfunction triggered by one or more precipitating factors, including acute illness, medication effects, or metabolic disturbances [2, 3]. For clinical characterization, delirium is commonly categorized into motor subtypes: hyperactive, hypoactive, and mixed forms [4]. Hyperactive delirium is characterized by restlessness, agitation, and, in some cases, aggressive behavior, whereas hypoactive delirium is characterized by lethargy, reduced motor activity, and social withdrawal [5]. Mixed forms fluctuate between these states [6].

Delirium constitutes a medical emergency because it is paramount to quickly identify and treat the underlying health issue as a precipitating factor. Furthermore, it is associated with significant short-, medium-, and long-term adverse outcomes. These include increased risks of falls, prolonged functional decline, persistent cognitive impairment, institutionalization, and mortality [7, 8]. Beyond individual complications, delirium imposes a considerable emotional burden on relatives and healthcare professionals [9, 10] and is associated with a substantial economic impact [11].

Dementia is one of the strongest predisposing risk factors for delirium [12]. When delirium occurs in individuals with preexisting dementia, the condition is referred to as delirium superimposed on dementia (DSD) [13]. Delirium and dementia share overlapping clinical features and have a complex bidirectional relationship. Delirium may accelerate cognitive decline and unmask previously undiagnosed dementia, whereas dementia increases susceptibility to delirium in the presence of acute stressors [14]. Consequently, detecting delirium in individuals with dementia is particularly challenging, as acute changes must be distinguished from preexisting cognitive impairment and fluctuating behavioral symptoms [15]. Nursing homes represent a high-risk setting for DSD because of the high prevalence of dementia, multimorbidity, polypharmacy, and frailty [16]. The epidemiological literature on delirium in nursing home populations is heterogeneous, with reported prevalence estimates ranging from 1.0% to 57.9% and a pooled prevalence estimate of 18.8% (95% CI: 7.4%–40.2%) [17]. This variability likely reflects differences in delirium assessment instruments, diagnostic classification approaches, and resident characteristics, particularly the proportion of participants with dementia. However, prevalence estimates also vary considerably among residents with dementia, ranging from 8% to 70.3% for DSD [18–20]. Differences in assessment approaches, including the use of heterogeneous or nonvalidated instruments, may contribute to this variability [18]. Prospective incidence data are even more limited. Only a few studies have quantified incident delirium in nursing home residents [18, 21–26]. In one study, the incidence rate was 3.4 per 100 person-weeks among residents with a prior diagnosis of dementia, compared with 0.8 per 100 person-weeks among residents without dementia [18]. Evidence on the duration, recurrence patterns, and long-term functional trajectories following delirium in residents with dementia remains limited. Accordingly, the National Institute for Health and Care Excellence (NICE) guidelines emphasize the need for large prospective cohort studies to clarify long-term outcomes in this high-risk population [27].

The Delirium Superimposed on Dementia in Residents of Nursing Homes (DeliDem-NH) study therefore aims to determine the prevalence and incidence of potential delirium among residents with dementia in nursing homes. Because the study relies on standardized screening instruments rather than physician-confirmed diagnostic assessments according to ICD-11 or DSM-5-TR criteria, cases identified through screening are referred to as “potential delirium.” In addition, the study examines associated risk factors, the duration and trajectory of delirium episodes, and their impact on cognitive and functional outcomes as well as mortality. Differences in physical activity between residents with and without potential delirium are explored, and current nursing and medical care processes related to delirium prevention and management are described.

## Methods

### Study design

The DeliDem-NH study is a prospective, multicentre cohort study with repeated measures. Data collection is planned from April 2026 to January 2028. Approximately 300 residents from 20 nursing homes (with an average of 15 participants per nursing home) with a documented diagnosis of dementia will be followed for 24 weeks, with an additional follow-up at week 36.

### Setting

Data collection will take place in nursing homes in northwest Germany, specifically in the regions of Oldenburg, Osnabrück, and Münster. Nursing homes with and without specialized gerontopsychiatric services and from both private and nonprofit providers will be eligible. For international comparability, a “nursing home” is defined as a facility providing 24-hour functional support for individuals requiring assistance with activities and/or instrumental activities of daily living, and who have identified health care needs, and where qualified nursing staff are available on site [28].

### Participants

Eligible participants are permanent residents of participating nursing homes with: (1) a documented diagnosis of dementia, (2) a Dementia Screening Scale score > 2 [29], and (3) informed consent from the legal representative. Exclusion criteria are severe receptive aphasia, deafness or extreme hearing impairment not compensable with hearing aids, and terminal status. Terminal status will be assessed by nursing home staff using an adapted “surprise question” [30]: whether *the staffmember would be surprised if the resident died within the next four weeks*.

### Recruitment

Nursing homes will be approached through regional networks and by direct email or telephone contact. Interested facilities will receive written study information and may request on-site or remote information sessions. To facilitate recruitment and retention, participating nursing homes will receive modest financial compensation for the effort associated with each completed screening. Each participating nursing home will identify residents meeting the predefined inclusion criteria at baseline and continuously throughout the recruitment period, including newly admitted residents. For all potentially eligible residents, basic pseudonymized information will be documented, including age, sex, documented dementia diagnosis, and, where available, type of dementia. This information will be forwarded to the study team and will be used to assess potential selection bias and nonresponse by comparing participating residents with eligible nonparticipants, using aggregated or pseudonymized data where feasible. If more than 15 eligible residents are identified, a computer-generated random selection will be performed at the facility level, and the first 15 residents will be selected. If 15 or fewer eligible residents are available, all will be selected.

The nursing home will request permission from the legal representatives of the selected residents to share residents’ names and contact details with the study team. Subsequently, the study team will contact the representatives to obtain proxy informed consent.

If fewer than 15 eligible residents are available at the time of initial recruitment, newly admitted residents will be screened and consecutively invited until the target of 15 participants per facility is reached. This rolling recruitment approach ensures that the predefined cluster size can be achieved whenever feasible.

If the target number of 15 participants cannot be reached in a participating nursing home, or if substantially fewer eligible residents than expected are available, additional participants from other participating nursing homes will be recruited.

### Sample size calculation

A total sample of 300 participants across approximately 20 clusters (mean sample size per cluster = 15) will be selected to allow estimation of the prevalence and incidence of potential delirium in residents with dementia while accounting for clustering at the facility level. A cluster-adjusted sample size approach was applied [31] because delirium occurrence may be influenced by facility-level factors.

Intraclass correlation coefficients (ICCC) of 0.10 for the prevalence estimation and 0.30 for the incidence estimation were assumed, similar to those reported in McCusker et al. [18]. Assuming a prevalence of 0.30, a cluster adjusted 95% confidence interval (CI) would be 0.22–0.38 based on all 300 participants. If a cumulative incidence of 0.30 in 6 months is assumed, a cluster adjusted 95% CI would be 0.18–0.42 based on only 10 participants per home without DSD at baseline (*n* = 200 in total). However, this incidence estimate does not account for variation in residents’ observation times (primarily due to death) and may, therefore, underestimate the true incidence. It was used solely for sample size calculation.

To obtain an initial estimate of the sample size required to detect differences between participants with and without delirium, we used malnutrition as an exemplary outcome. Based on previous findings, malnutrition can be assumed to be present in approximately 18% of nursing home residents without delirium and in 47% of residents with delirium [32]. In a cluster-randomized trial with *n* = 20 clusters of mean size 15, an ICCC of 0.10, and a power of 80–85%, we would be able to detect differences between the delirium and non-delirium groups of 23% versus 47% regarding malnutrition using a cluster adjusted chi-square test [31]. This is a conservative estimate of power, because the DeliDem-NH study is an observational study and not a cluster randomized trial.

### Study flow and measurement schedule

**Fig. 1:**
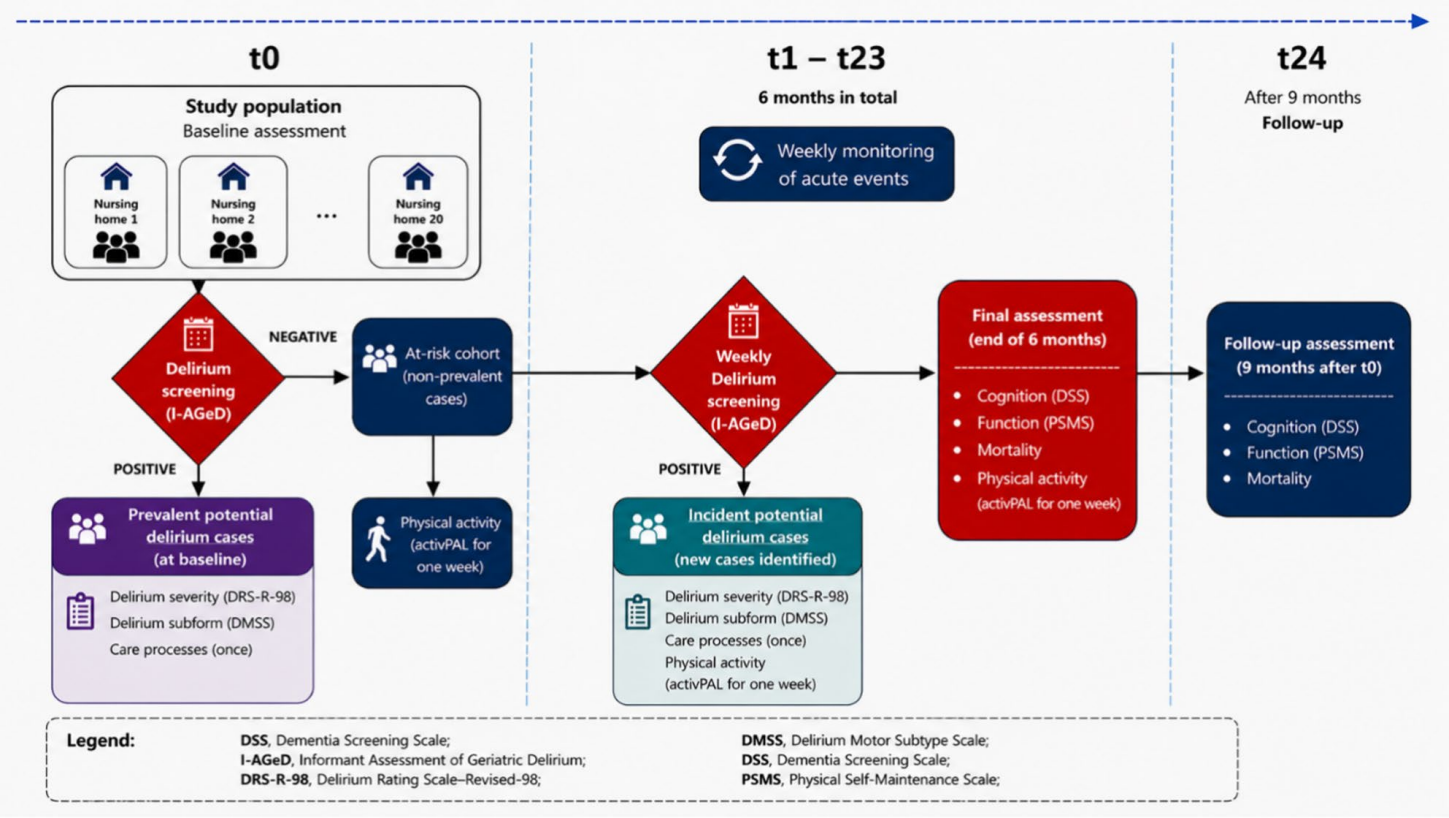
Data Collection Procedure of the DeliDem-NH Study

Assessments are conducted at baseline (t0), weekly from weeks 1 to 22 (t1–t22), at the end of the 24-week observation period (t23), and at a follow-up three months after completion of the observation period at week 36 (t24). The interval between baseline and the final assessment is 24 weeks (six months) (Fig. 1).

#### Baseline t0

At baseline (t0), all participants will undergo a comprehensive assessment, including screening for potential delirium. Participants with a positive delirium screening result at baseline are the prevalence sample and will not be followed longitudinally and will not contribute to incidence or prognostic analyses. Participants without evidence of delirium will form the at-risk cohort, which will be followed prospectively.

In the prevalent cases identified at baseline, delirium severity and the specific delirium subtype will be assessed. We will also assess nursing and medical processes by questionnaire in the prevalent cases.

Physical activity will be measured at baseline only in the at-risk cohort.

Structural characteristics of the participating nursing homes (e.g., size, number of beds, specialization, and organizational characteristics) will be collected once at t0 for descriptive purposes.

#### Weekly assessments t1-t23

Weekly assessments (t1-t23) will be conducted to identify incident cases of potential delirium. These assessments will take place during weekly visits (Monday to Friday, during the daytime) in the participating nursing homes. In the event of a positive delirium screening, symptom severity and motor subtype will be assessed. Regardless of screening results, all participants will receive brief weekly visits by members of the study team, if feasible. Additionally, acute events occurring during the previous week (falls, infections, catheter or tube insertions, or changes in medication regimens), as well as hospital or emergency department admissions will be recorded. In the week following the first identification of potential delirium, physical activity will be measured. Nursing and medical care processes related to delirium management will be assessed once, after the first occurrence of potential delirium.

If assessments cannot be performed temporarily, for example, due to hospitalization or other absence, this will be documented, and participants will be reassessed upon return to the nursing home. Follow-up will be discontinued in of the event of withdrawal of consent, moving to a different nursing home, or death. At the final assessment (t23), cognitive function and functional status will be reassessed using the same instruments applied at baseline. Vital status will also be recorded. In addition, physical activity will be measured for one week.

#### Follow-up t24

At follow-up (t24), three months after the final assessment, cognitive function, functional status, and vital status will be reassessed using the baseline instruments.

### Variables and measurements

#### Primary outcome

The primary endpoint of the study is the occurrence of potential delirium. Potential Delirium will be assessed using the *Informant Assessment of Geriatric Delirium* (I-AGeD) [33], an informant-based screening instrument. The I-AGeD consists of ten dichotomous (yes/no) items that capture acute changes in behavior and cognition: *“I do not recognize him/her as their usual self”*,* (2) “I often have to repeat things to get his/her attention”*,* (3) “He/she is less alert and/or appears to be drowsy during the daytime”*,* (4) “He/she has little spontaneous movement and hardly moves the upper limbs”*,* (5) “He/she is often awake at night and sleepy during the day”*,* (6) “He/she has recently become more forgetful”*,* (7) “When the conversation stops*,* his/her eyes close”*,* (8) “He/she is difficult to awaken”*,* (9) “He/she is combative and struggles to get free”*, and *(10) “He/she says strange things that don`t make any sense”*. The I-AGeD contains dichotomous “yes” and “no” answers. A total score of ≥ 4 indicates potential delirium. The instrument allows rapid assessment of acute changes without requiring active participation from residents. It is, therefore, suitable for weekly screening in nursing home settings, where it is recommended for delirium monitoring [34, 35]. For the calculation of the I-AGeD total score, missing item values will be handled according to a predefined imputation rule. If only one of the ten items is missing, the missing value will be replaced by the response category, either “yes” or “no”, that occurs more frequently among the remaining nine items. If more than one item is missing, the total score will be considered missing and will not be calculated for that assessment.

#### Additional outcomes and measures

##### Delirium severity

The severity of potential delirium symptoms is assessed using the *Delirium Rating Scale–Revised-98* (DRS-R-98) [36]. The DRS-R-98 is a clinician-rated instrument comprising 13 severity items and 3 diagnostic items (scored 0–3). It can be completed by psychiatrists, other physicians, nurses, or psychologists with appropriate clinical training in the assessment of psychiatric phenomena [36]. The 13 severity items (sleep–wake cycle disturbance, perceptual disturbances and hallucinations, delusions, lability of affect, language, thought process abnormalities, motor agitation, motor retardation, orientation, attention, short-term memory, long-term memory, and visuospatial ability) yield a maximum total score of 39 points and are used in this study to capture symptom severity. The DRS-R-98 includes several tests that require active participation from the resident. However, it can also be applied as an informant- or proxy-based assessment tool. A study is currently investigating the interrater reliability of the German DRS-R-98 in people with dementia when used by nurses in nursing homes [37].

##### Delirium motor subtype

For motor subtyping of potential delirium cases, the *Delirium Motor Subtype Scale* (DMSS) is applied [38]. The proxy-based DMSS consists of 11 items (4 hyperactive and 7 hypoactive) to classify hyperactive, hypoactive, or mixed delirium. Hyperactive delirium is defined by ≥ 2 positive hyperactive items; hypoactive delirium requires a positive item 5 or 6 plus at least one positive among items 7–11. If the criteria for both types are met, a mixed subtype is classified; if neither is met, no motor subtype is assigned. The German version demonstrates good psychometric properties [38].

#### Mortality

Vital status (alive or deceased) will be recorded weekly, at the end of the observation period (t23) and at follow-up (t24) for all participants.

#### Physical activity

Objective physical activity is measured using the triaxial accelerometer activPAL (PAL Technologies Ltd., Glasgow, UK). The device is small and lightweight (23.5 mm wide, 43 mm long, 5 mm thick, and weighing 9.5 g) and is attached to the thigh with a medical-grade adhesive. It is worn continuously for seven days and differentiates between sitting/lying and upright (standing/walking) positions. The device has previously been used in geriatric inpatients with delirium [39]. The primary parameter is standing/stepping time.

#### Nursing and medical care processes

When potential delirium is identified, a standardized questionnaire is sent to the responsible general physician to document diagnostic and therapeutic procedures (e.g., history taking, laboratory and imaging tests, medication changes, fluid administration, nonpharmacological interventions, and hospital transfers). Another questionnaire captures preventive measures, delirium management by nursing staff (e.g., environmental adjustments, orientation aids, and communication with relatives). This questionnaire is filled out by the nurses. Since no validated instruments exist for this purpose, the questionnaires were developed by the multidisciplinary study team and aligned with the current German S3 guideline on delirium in older age [40].

An overview of all dependent and independent variables, assessment tools, and measurement time points is provided in Table 1.

**Table 1:** Overview of study variables, assessment instruments, and timing of data collection

Domaine/Instrument	Baseline t0	Weekly assessments t1-t22	Final examination t23	Follow-up t24
Potentoaö delirium (I-AGeD) [35]	**A**,** B**	**B**	**B**	
Delirium severity (DRS-R-98) [36]	**A**	**B** ^**a**^	**B** ^**a**^	
Delir Motor subtype (Delirium Motor Subscale, DMSS) [38]	**A**	**B** ^**a**^	**B** ^**a**^	
Cognition (Dementia Screening Scale, DSS) [29]	**A**,** B**		**B**	**B**
Function (Physical Self Maintenance Scale, PSMS) [41]	**A**,** B**		**B**	**B**
Pain (Pain Assessment in Advanced Dementia Scale, PAINAD) [42]	**A**,** B**			
Neuropsychiatric symptoms (Neuropsychiatric Inventory Questionnaire, NPI-Q) [43]	**A**,** B**			
Nutritional status (Mini Nutritional Assessment – Short Form, MNA-SF) [44]	**A**,** B**			
Depression (Cornell Scale for Depression in Dementia) [45]	**A**,** B**			
Physical activity (ActivPAL)	**B**	**B** ^**a**^	**B**	
History of falls (past week)	**A**,** B**	**B**	**B**	
Positive history of delirium (past 6 months)	**A**,** B**			
Hospital admission (past 6 months)	**A**,** B**			
Hospital admission (past week)	**A**,** B**	**B**	**B**	
Transfer to emergency department (past 6 months)	**A**,** B**			
Transfer to emergency department (past week)	**A**,** B**	**B**	**B**	
Authorization for the use of physical restraints	**A**,** B**			
Feeding tube	**A**,** B**			
New placement of feeding tube		**B** ^**b**^	**B** ^**b**^	
Removal of feeding tube		**B** ^**c**^	**B** ^**c**^	
Urinary catheter	**A**,** B**			
New placement of urinary catheter		**B** ^**b**^	**B** ^**b**^	
Removal of urinary catheter		**B** ^**c**^	**B** ^**c**^	
Infection (past week)	**A**,** B**	**B**	**B**	
Reduced fluid intake (past week)	**A**,** B**	**B**	**B**	
Medical diagnoses Types of dementia, Parkinson’s disease, Depression, Hypertension, Atrial fibrillation, Chronic heart failure, Peripheral vascular disease, Diabetes mellitus, COPD, Chronic kidney disease, Liver cirrhosis, schizophrenia, bipolar disorder, and addictive disorders, History of stroke, Anemia	**A**,** B**			
Care level	**A**,** B**			
Hearing impairment	**A**,** B**			
Visual impairment	**A**,** B**			
Age	**A**,** B**			
Sex	**A**,** B**			
Length of stay in the facility	**A**,** B**			
Marital status	**A**,** B**			
Medication				
Medication name Dosage strength and unit Pharmaceutical form	**A**,** B**			
Mode of administration (REGULAR / PRN)	**A**,** B**			
Changes to the medication regimen	**A**,** B**	**B**	**B**	
Nursing processes Medical diagnostic procedures	**A**	**B** ^**a**^	**B** ^**a**^	
Mortality		**B**	**B**	**B**

A = Prevalent delirium cases at baseline: residents screening positive on the I-AGeD at baseline (t0)

B = At-risk cohort (non-prevalent cases): residents screening negative on the I-AGeD at baseline (t0)

B^a^ = Assessment performed following a positive I-AGeD screening result

B^b^ = Assessment performed if no urinary catheter or feeding tube was present at baseline or at the previous assessment

B^c^ = Assessment performed if a urinary catheter or feeding tube was present at baseline or at the previous assessment

t0–t24 = Time points of data collection

#### Medication

At t0, medication will be assessed. In line with previous research studies in older persons, including nursing home residents [46, 47], medication data will be obtained from photographs of the medication plans allowing a distinction between regular and as-needed medication ). Later, these photographs will be used to enter medication data into a prespecified database, where the pharmaceutical central number and the anatomical therapeutic chemical (ATC) classification system can be linked. Medications (e.g., regarding anticholinergic and sedative properties) will later be selected based on the ATC code.

### Raters and training

All assessments will be conducted by two research associates and two study nurses (hereafter referred to as raters). All raters hold qualifications in the medical or nursing field, and the research staff have completed a university-level degree. Raters are expected to have clinical experience in caring for individuals with dementia or delirium, preferably both. All raters will undergo clinical training in the Geriatrics Department at Klinikum Oldenburg. This training includes participation in ward rounds and hands-on practice with the instruments used in the study, with the goal of enabling independent administration. Finally, the raters will conduct cognitive tests and delirium screenings independently under the supervision of an expert. In addition, simulation sessions will be conducted through the simulation person program of Carl von Ossietzky University Oldenburg to further consolidate the application of the study instruments.

Nursing home staff and the individuals responsible for the project will receive a 90-minute training session on delirium, the assessment instruments used, and the study procedures.

### Data management and data protection

Data will be collected using a custom-built REDCap database hosted at Carl von Ossietzky Universität Oldenburg. Data entry will be performed either via the REDCap Mobile App on university-owned, password-protected tablets or through the REDCap website using a university-owned laptop. Access to the database will be role-based and restricted to authorized study personnel [48, 49].

All data will be processed in accordance with applicable data protection regulations, including the EU General Data Protection Regulation. The pseudonymization key will be deleted after completion of the data analyses unless further retention is legally required. Only anonymized, aggregated results will be published.

Study data will be checked for plausibility at Carl von Ossietzky Universität Oldenburg. Afterwards, encrypted pseudonymized data will be transferred to mediStatistica for statistical analysis.

### Statistical analysis

Baseline characteristics of nursing homes and participating residents will be described using frequencies and proportions for categorical variables and means with standard deviations or medians with interquartile ranges for continuous variables. These descriptive statistics will be presented without cluster adjustment.

All primary outcome analyses will account for clustering at the facility level.

#### Prevalence analysis

Delirium prevalence at baseline will be estimated for the total study population and stratified by age and sex. Cluster-adjusted 95% confidence intervals will be calculated using the estimated intraclass correlation coefficients.

#### Incidence analysis

Incidence rates will be calculated at the cluster level as the mean number of delirium cases per 100 person-weeks in each cluster, with corresponding 95% confidence intervals assuming approximate normality of cluster-specific incidence rates. Person-weeks are counted until the end of observation at the first occurrence delirium, censoring or death. Reasons for censoring might include hospitalization until death or the end of the study.

Additionally, individual-level incidence rates (cases per 100 person-weeks) will be estimated assuming a Poisson distribution without cluster adjustment to facilitate comparison with previous literature.

To account for varying follow-up times and competing risks, cumulative incidence curves for delirium, death, and hospitalization will be estimated, considering death and hospital admission as competing events.

#### Analysis of predisposing and precipitating factors

To investigate potential risk factors for the prevalence of delirium at baseline, multiple mixed logistic regression models will be fitted to the whole study population with prevalence as the dependent variable and predefined candidate variables as independent variables, including cluster adjustment by random effects. Candidate variables will be defined a priori based on clinical relevance and the literature and will include demographic characteristics, baseline clinical and functional status, cognitive impairment, neuropsychiatric symptoms, nutritional status, pain, depression, sensory impairments, care level, and comorbidities, as well as prior health events such as a history of delirium, falls, hospital admissions, or emergency department transfers.

To investigate potential risk factors for the incidence of delirium during the six months after baseline, multiple frailty Cox regression models will be fitted in the at-risk cohort, with time to delirium (censored if last observation without delirium) as dependent variable and predefined candidate variables as independent variables. These will include both baseline characteristics and events occurring during the observation period (e.g., falls, hospital or emergency department transfers, placement of catheters). Variables derived from repeated weekly assessments will be modeled as time-varying covariates where appropriate. Cluster adjustment will be performed using a frailty term.

#### Missing values

Residents in the at-risk cohort will be analyzed with respect to incident delirium between baseline and the first occurrence of delirium or death. In cases of interim missing values before the last observation sensitivity analyses involving the imputation of missing values will be discussed, e.g., multiple imputation if feasible. The primary outcome, secondary outcomes, potential risk factors, confounders and baseline variables will be included in the imputation model. Missing values may occur for several reasons, including temporary absence from the nursing home due to hospital admission, residents being away during holiday periods, or temporary restrictions in data collection related to infectious disease outbreaks within the facility.

#### Prognostic analyses (exploratory)

For prognostic analyses, residents with delirium will be classified into two groups:

1. Remitted delirium (residents with one delirium episode followed by resolution and no recurrence during follow-up), and
2. Persistent or recurrent delirium (residents with delirium persisting from the first time point of detection until the end of their weekly measurement period without documented resolution, or residents with a new delirium episode after a resolved episode).

A delirium episode is defined as one or more consecutive positive I-AGeD assessments. Resolution of delirium is defined as at least two consecutive negative assessments to account for the fluctuating nature of delirium. A recurrent episode is defined as a new positive I-AGeD assessment following a resolved episode.

Primarily only participants without missings of I-AGeD assessments over the period from t0 to t23 will be analyzed describing baseline variables and the time course of time-varying variables separated for both subgroups. Where feasible, confidence intervals or statistical tests will be cluster-adjusted.

Participants who are hospitalized or deceased before final or follow-up assessment will be analyzed separately in an explorative, descriptive manner.

#### Physical activity analyses

To examine differences in physical activity, upright time (minutes per day) measured via accelerometry will be analyzed in the at-risk cohort. Linear mixed models will be estimated with physical activity as the dependent variable and delirium status (time-dependent), time since baseline, age, and sex as fixed effects. Random effects will account for repeated measurements within individuals and clustering within nursing homes. Physical activity is measured for all participants in the at-risk cohort at baseline (t0) and will be assumed to be constant until the next measurement at the first occurrence of delirium or at t23.

If model convergence is not achieved due to the limited sample size, activity levels in residents with delirium will be descriptively compared with matched controls (matched on age, sex, and, where possible, cluster membership).

#### Analysis of nursing and medical care processes

Data on nursing and medical care processes will be analyzed descriptively using relative frequencies. Differences between male and female residents will be explored. Data from prevalent delirium cases and the at-risk cohort will be analyzed jointly for these process-related outcomes. No interim analyses are planned.

#### Ethics

The DeliDem-NH study will be conducted in accordance with the Declaration of Helsinki and relevant national regulations. Ethical approval has been granted by the Medical Ethics Committee of the University of Oldenburg (approval number 2025–260). Written informed consent will be obtained from the residents’ legal representatives prior to study participation. Residents’ presumed wishes and current assent or dissent will be respected throughout the study. If raters observe verbal or nonverbal signs indicating that a resident does not wish to participate in or continue an assessment, the assessment will be discontinued. In such cases, resident-related information required for the study will, where possible, be obtained with the assistance of nursing staff rather than through direct resident assessment.

Additional safeguards will apply to the sensor-based measurement of physical activity. If the legal representative declines the use of the sensor, physical activity data will not be collected, but the resident may still participate in all other study components. Concerns raised by relatives or nursing staff will be considered before the sensor is attached. The sensor will not be attached if there are concerns that the adhesive patch may cause skin irritation, discomfort, or harm. If the sensor causes distress, discomfort, or any other adverse effect during the monitoring period, it will be removed immediately.

## Discussion

Epidemiological research on DSD in nursing homes remains limited and methodologically heterogeneous. Previous studies differ substantially in diagnostic criteria, assessment intervals, and measurement instruments, and often exclude residents with moderate to severe dementia despite their particularly high risk. The DeliDem-NH study addresses these research gaps through a prospective longitudinal design with frequent, standardized assessments in real-world settings across a large number of nursing homes. In contrast to many previous studies, residents with advanced dementia are explicitly included, unless they have severe receptive aphasia, deafness, or extreme hearing impairment that cannot be compensated for with hearing aids. Weekly assessments are intended to better capture the fluctuating nature of delirium and to improve detection of transient and recurrent episodes. We will use validated instruments such as the I-AGeD and the DRS-R-98, which assess core delirium domains including attention, higher cognitive function, and circadian disturbances [34, 50]. Conducting the study within routine care enhances ecological validity and leverages staff familiarity with residents’ baseline cognitive and functional status. Combining this local knowledge with structured external assessments can improve detection of subtle acute changes.

An additional strength compared with prior research is the integration of objective physical activity monitoring using thigh-worn accelerometry. This approach may provide complementary information on psychomotor alterations and improve the detection of hypoactive and mixed delirium subtypes, which are frequently underrecognized in routine care. Furthermore, continuous monitoring across daytime and nighttime periods may enable the identification of disturbed activity patterns and restless behavior that may provide additional clinically relevant indicators of delirium. The structured documentation of nursing and medical care processes following suspected delirium also allows for the evaluation of real-world management strategies and their alignment with guideline recommendations.

### Methodological considerations and anticipated challenges

For pragmatic reasons, case detection relies on validated screening instruments and the concept of potential delirium rather than universal physician-confirmed DSM or ICD criteria. Some degree of misclassification is therefore possible. However, cases identified as potential delirium are communicated to the responsible general practitioner for further clinical evaluation and appropriate management. Although weekly assessments are relatively frequent, short or rapidly fluctuating episodes occurring between visits may be missed. Data collection is limited to daytime hours and may therefore underestimate nocturnal symptoms. Proxy-based instruments and informant reports are necessary but introduce potential measurement bias and heterogeneity, particularly when different staff members complete assessments for the same resident at different times. In advanced dementia, differentiating delirium from progression of the underlying neurocognitive disorder can be inherently difficult. Expected attrition due to morbidity and mortality will reduce person-time at risk and may cause bias, reduce statistical power and limit generalizability. Furthermore, incomplete data in the follow-up because of hospital stays might cause bias and loss of power, even if missing data are partially addressed through imputation. Analyses of physical activity data might be limited by missing values arising from technical problems or loss of the sensors. Finally, heterogeneity across participating facilities, participating facilities, for example in staffing levels and organizational structures, requires careful analytical control through cluster adjustment.

### Implications for research and practice

The expected findings have immediate practical relevance. Detailed epidemiological data on frequency, temporal patterns, and associated factors of delirium in long-term care can inform targeted, resource-sensitive prevention and management strategies. Such approaches might include structured screening procedures, staff education, medication review, and context-adapted nonpharmacological interventions that enable nursing staff to detect delirium earlier, initiate appropriate in-facility measures, and potentially reduce avoidable hospital transfers. Scientifically, DeliDem-NH will refine risk profiles, improve understanding of care processes before, during, and after delirium episodes, and help to identify subgroups most likely to benefit from specific preventive or therapeutic measures.

## Data Availability

No datasets were generated or analysed during the current study.

## Abbreviations

DSD: Delirium Superimposed on Dementia
DeliDem-NH: Delirium Superimposed on Dementia in Residents of Nursing Homes
DRS-R-98: Delirium Rating Scale-Revised-98
DMSS: Delirium Motor Subtype Scale
I-AGeD: Informant Assessment of Geriatric Delirium
ICCC: Intraclass Correlation Coefficients
